# Unhealthy lifestyles, environment, well-being and health capability in rural neighbourhoods: a community-based cross-sectional study

**DOI:** 10.1186/s12889-021-11661-4

**Published:** 2021-09-06

**Authors:** Anabela Marisa Azul, Ricardo Almendra, Marta Quatorze, Adriana Loureiro, Flávio Reis, Rui Tavares, Anabela Mota-Pinto, António Cunha, Luís Rama, João Oliveira Malva, Paula Santana, João Ramalho-Santos, HeaLIQs4Cities consortium, António Cunha, André Pardal, Eugénia Peixoto, Diana Guardado, Marieke Zwaving, Eduardo Briones Pérez De La Blanca, Roel A. van der Heijden, Ruth Koops Van’t Jagt, Daan Bultje, João Malva, Flávio Reis, Luís Rama, Manuel Veríssimo, Ana Teixeira, Margarida Lima, Lèlita Santos, Filipe Palavra, Pedro Ferreira, Anabela Mota Pinto, Paula Santana, Ricardo Almendra, Adriana Loureiro, Inês Viana, Marta Quatorze, Anabela Marisa Azul, João Ramalho-Santos, Catharina Thiel Sandholdt, Maria Kristiansen

**Affiliations:** 1 Center for Neuroscience and Cell Biology (CNC), University of Coimbra, 3004-504 Coimbra, Portugal; 2 Center for Innovative Biomedicine and Biotechnology (CIBB), University of Coimbra, 3030-789 Coimbra, Portugal; 3 University of Coimbra, Institute for Interdisciplinary Research (IIIUC), 3030-789 Coimbra, Portugal; 4 Centre of Studies in Geography and Spatial Planning (CEGOT), Faculty of Arts and Humanities, Colégio de São Jerónimo, University of Coimbra, 3004-530 Coimbra, Portugal; 5 Department of Geography and Tourism, Faculty of Arts and Humanities, Colégio de São Jerónimo, University of Coimbra, 3004-530 Coimbra, Portugal; 6 Coimbra Institute for Clinical and Biomedical Research (iCBR), Faculty of Medicine, University of Coimbra, 3030-789 Coimbra, Portugal; 7 Institute of Pharmacology & Experimental Therapeutics, Faculty of Medicine, University of Coimbra, 3030-370 Coimbra, Portugal; 8 Clinical Academic Center of Coimbra (CACC), 3030-370 Coimbra, Portugal; 9 CIMAGO-Center for Research in the Environment, Genetics and Oncobiology, Faculty of Medicine, University of Coimbra, Coimbra, Portugal; 10 IPN-Laboratory of Automatics and Systems, Pedro Nunes Institute, 3030-199 Coimbra, Portugal; 11 Ageing@Coimbra, EIP on AHA Reference Site, Coimbra, Portugal; 12 Faculty of Sport Sciences and Physical Education, University of Coimbra, 3040-256 Coimbra, Portugal; 13 Department of Life Sciences (DCV), Faculty of Sciences and Technology, University of Coimbra, 3000-456 Coimbra, Portugal

**Keywords:** Non-communicable diseases, Healthy lifestyles, Health loss, Health capability, Qualitative driven mixed-methods, Participatory community-based research, Built environment, Natural environment, Rural areas

## Abstract

**Background:**

Non-communicable diseases are a leading cause of health loss worldwide, in part due to unhealthy lifestyles. Metabolic-based diseases are rising with an unhealthy body-mass index (BMI) in rural areas as the main risk factor in adults, which may be amplified by wider determinants of health. Changes in rural environments reflect the need of better understanding the factors affecting the self-ability for making balanced decisions. We assessed whether unhealthy lifestyles and environment in rural neighbourhoods are reflected into metabolic risks and health capability.

**Methods:**

We conducted a community-based cross-sectional study in 15 Portuguese rural neighbourhoods to describe individuals’ health functioning condition and to characterize the community environment. We followed a qualitatively driven mixed-method design to gather information about evidence-based data, lifestyles and neighbourhood satisfaction (incorporated in eVida technology), within a random sample of 270 individuals, and in-depth interviews to 107 individuals, to uncover whether environment influence the ability for improving or pursuing heath and well-being.

**Results:**

Men showed to have a 75% higher probability of being overweight than women (*p*-value = 0.0954); and the reporting of health loss risks was higher in women (RR: 1.48; *p*-value = 0.122), individuals with larger waist circumference (RR: 2.21; IC: 1.19; 4.27), overweight and obesity (RR: 1.38; *p*-value = 0.293) and aged over 75 years (RR: 1.78; *p*-value = 0.235; when compared with participants under 40 years old). Metabolic risks were more associated to BMI and physical activity than diet (or sleeping habits). Overall, metabolic risk linked to BMI was higher in small villages than in municipalities. Seven dimensions, economic development, built (and natural) environment, social network, health care, demography, active lifestyles, and mobility, reflected the self-perceptions in place affecting the individual ability to make healthy choices. Qualitative data exposed asymmetries in surrounding environments among neighbourhoods and uncovered the natural environment and natural resources specifies as the main value of rural well-being.

**Conclusions:**

Metabolic risk factors reflect unhealthy lifestyles and can be associated with environment contextual-dependent circumstances. People-centred approaches highlight wider socioeconomic and (natural) environmental determinants reflecting health needs, health expectations and health capability. Our community-based program and cross-disciplinary research provides insights that may improve health-promoting changes in rural neighbourhoods.

**Supplementary Information:**

The online version contains supplementary material available at 10.1186/s12889-021-11661-4.

## Background

Non-communicable diseases (NCD) are the leading causes of health loss globally, accounting for 91% of deaths and almost 87% of disability-adjusted-life-years (DALYs) in Europe [1], in part due to unhealthy diets and lifestyles [2, 3]. A systematic analysis for the Global Burden of Disease [4], undertaken by the World Health Organisation (WHO) and the Institute for Health, Metrics and Evaluation (IHME) highlight three metabolic risks among the five leading risks of DALYs worldwide: i) high systolic blood pressure (SBP), ii) high fasting plasma glucose and iii) body-mass index (BMI). In parallel, in 2019, a large-scale study including more than 112 million adults across urban and rural neighbourhoods estimated that BMI increased 2.1 kg/m^2^ in both women and men in rural neighbourhoods over the past three decades; suggesting that the rising of rural BMI is currently the main health risk factor in adults [5].

Health loss risks in rural neighbourhoods may be amplified by wider determinants of health and well-being such as the geographic and historical factors across economic and socio-cultural characteristics [6]. Places are living organisms that produce dynamics, generate environments and create societies [7, 8] They are a set of multiple, complex and overlapping environments that support life (e.g., home, social relationships, communities and neighbourhoods) [9]. The exposure to positive or negative environments, that occur in particular geographic locations, influence human health and well-being throughout the course of life [10, 11]. Problems related with built, connective, and relational space present themselves when spatial planning and development models cannot be adjusted in face of a changing landscape, for instance, ageing phenomena [12]. A growing elderly population accentuates the ability to pursue health in place due to a combination of physical–cognitive and functional–social and psychological fragility [10–13].

Communities have a deep understanding of their surrounding environments enabling them to better assess external factors [13, 14] impacting health and the ability to make healthy choices. Comprehensive theories of health and social justice [15–19] intersect individual-level data and broader structural and environmental circumstances, for mapping the conditions that reflect health needs, health expectations and health capability gaps at both individual and community levels. In this way, Ruger’ health capability mode of 2010 [18] includes the capability to reduce/prevent the exposure to metabolic risks factors, to reduce DALYs and early mortality, to pursue healthy lifestyles, or to gain health-related knowledge, which is viewed both as an end for individuals (intrinsic motivation) but also as a driving force for encouraging changes at the community level, e.g., socioeconomic development, built and natural environment, or social cohesion, particularly in rural areas [20].

Self-management of NCD remains poorly implemented in rural neighbourhoods despite self-adherence to healthy lifestyles evidence reflected in self-ability to make balanced decisions [21, 22]. The community-based participatory research (CBPR) is a wide-ranging methodological approach that concedes the possibility of exploring gaps between what is expected and what is afforded and its interconnections and interdependencies [23], while evidence-based data can be helpful for assessing an individual’s health functionality. Therefore, we propose a qualitatively driven mixed-method design to assess unhealthy lifestyles of people living in rural neighbourhoods, which includes gathering evidence-based data about metabolic risks and health functionality and studying broader contextual determinants of health and well-being associated to place and neighbourhood. We ultimate expect to uncover health and well-being drivers in rural neighbourhoods, and determine whether community circumstances influence health capability at both the individual and community-level.

## Methods

### Study area, design and community setting

**Fig. 1:**
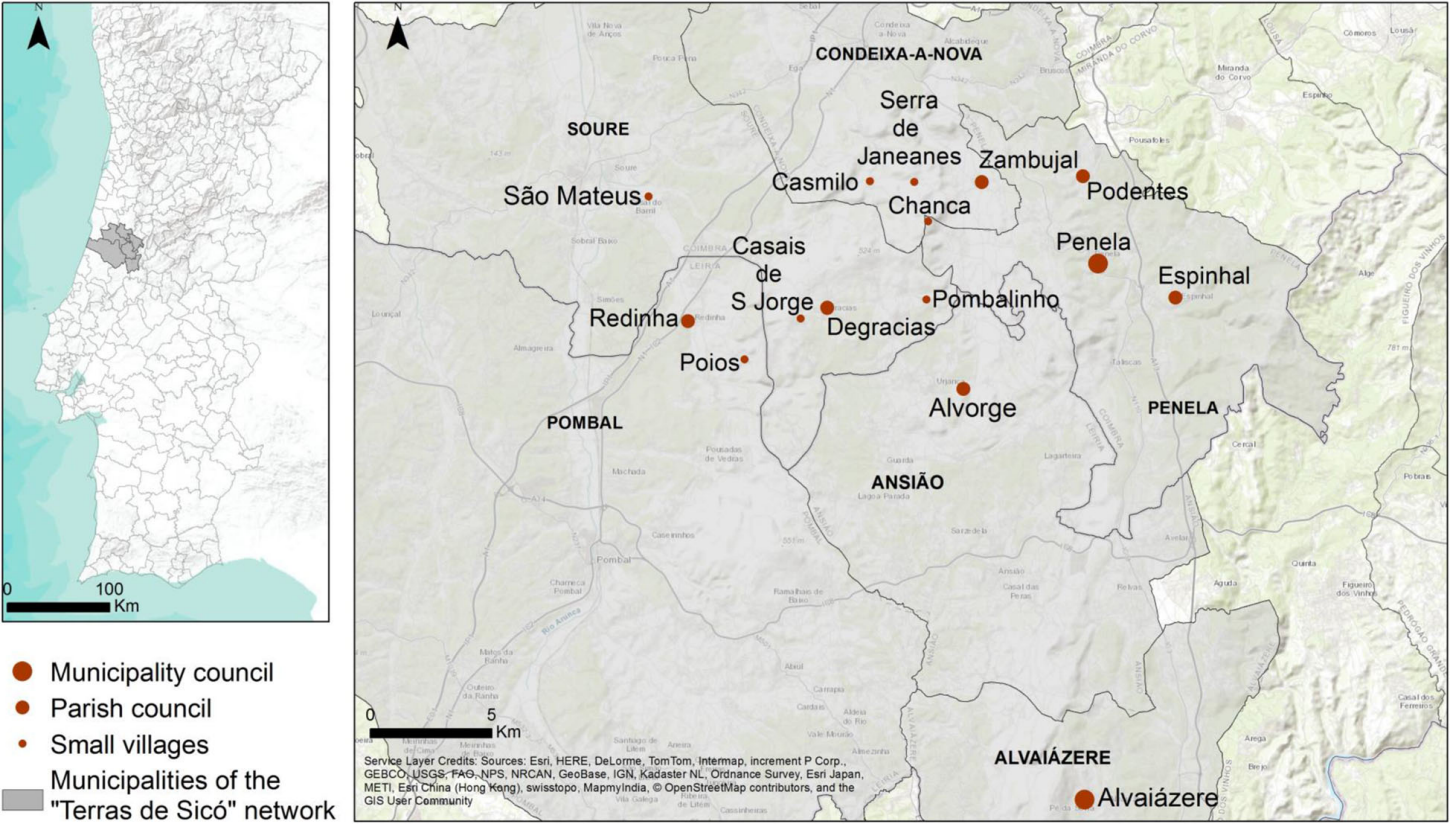
Location of rural neighbourhoods; basemap is provided by ESRI, available as part of the mapping platform ArcGIS Online

The cross-sectional study was conducted in 15 rural neighbourhoods from six municipalities in the Centre region of Portugal (Fig. 1), aiming at 1) assessing evidence-based data and describing lifestyles, 2) examining determinants of health and well-being in rural neighbourhoods, and 3) discuss how individuals’ conditions and population’ circumstances can contribute with a better understanding to improve health capability in rural neighbourhoods.

The selection of the rural neighbourhoods of the “*Terras de Sicó*” (*Lands of Sicó*) network (Sicó-network) was drawn on a CBPR approach. Given possible differences at the administrative level, which could influence local practices, we considered the three relevant levels of territory administrative structure: small villages, parish councils, and municipalities seats (hereinafter referred as municipality) (Fig. 1). According to the Portuguese National Statistics Institute, in 2011, 3879 individuals were living in the 15 rural neighbourhoods (Table 1), one third of the population was older than 64 years and with a high rate of limited literacy (e.g., the proportion of individuals that do not know how to read is almost the same as individuals with higher education); which are common characteristics in Portuguese rural areas [24].

**Table 1:** Demographic and social characteristics of the individuals by neighbourhoods’ type

	Small Village	Parish Council	Municipality	Total
	(*n* = 84)	(*n* = 112)	(*n* = 74)	(*n* = 270)
Age, years				
< 35	2 (2%)	3 (3%)	11 (15%)	16 (6%)
35–54	7 (8%)	20 (18%)	15 (20%)	42 (16%)
55–74	42 (50%)	48 (43%)	38 (51%)	128 (47%)
> 74	33 (39%)	41 (37%)	10 (14%)	84 (31%)
Sex				
Female	56 (67%)	64 (57%)	50 (68%)	170 (63%)
Male	28 (33%)	48 (43%)	24 (32%)	100 (37%)
Education				
None	24 (29%)	10 (9%)	5 (7%)	39 (14%)
1st cycle of basic education	48 (57%)	71 (63%)	29 (39%)	148 (55%)
2nd cycle of basic education	6 (7%)	9 (8%)	5 (7%)	20 (7%)
3rd cycle of basic education	–	9 (8%)	8 (11%)	17 (6%)
Secondary education	3 (4%)	10 (9%)	9 (12%)	22 (8%)
Higher Education	3 (4%)	3 (3%)	18 (24%)	24 (9%)
Employment status				
Unemployed	6 (7%)	1 (1%)	3 (4%)	10 (4%)
Employed	11 (13%)	32 (29%)	36 (49%)	79 (29%)
Student	–	1 (1%)	2 (3%)	3 (1%)
Unable to work	1 (1%)	–	2 (3%)	3 (1%)
Retired	65 (77%)	77 (69%)	30 (41%)	172 (64%)

Data are in (%), some percentages might not add to 100% due to option given to subjects of not answering

The study encompasses a qualitatively driven mixed-method design, that is, simultaneously, qualitative (QUAL; inductive theoretical drive) and quantitative (quan): QUAL+quan [25]: quan to describe and examine individuals’ health functioning condition (evidence-based data and lifestyles); QUAL to document how individuals experience their neighbourhood in terms of health and well-being [26], and to better understand which local circumstances influence the ability to adopt healthier lifestyles and to pursue health [18].

Our CBPR approach involved the local representatives from the Sicó-network (*n* = 20; among policymakers, local community members and stakeholders); advanced training students and young professionals (*n* = 13), from biomedical sciences, medicine and sports sciences; a trans-disciplinary research and innovation team (*n* = 18) involving researchers from life sciences, medical and health sciences, and social sciences, and developers of advanced technology for health monitoring and e-health services, including two international members of the HeaLIQs consortium and two members of the consortium Ageing@Coimbra. Two local consolidation meetings with local representatives of the Terras de Sicó network and the research and innovation team, held in two municipalities, Penela (May 28, 2019) and Alvaiázere (June 11, 2019), created the bases of the CBPR approach, and a roadmap for local itineraries and local community engagement. Triangulation between local representatives and researchers regarding the CBPR approach contributed to: better characterizing the demography in the 15 neighbourhoods; co-designing the community program adapted to each neighbourhood; co-constructing a health communication strategy and tailored healthy lifestyles-related messages for older adults with limited literacy; discussing the theoretical background [14–20] and the QUAL+quan methodology connecting with a questionnaire [27] incorporated in pre-existing eVida technology [28]; and training volunteer students and young professionals to operationalize translational research and participatory approaches with community engagement in neighbourhoods. Local representatives collaborated actively in the dissemination of the program via national/regional media (i.e., newspapers, radio, television and flyers), social media (i.e., Facebook) and institutional websites (e.g., Sicó-network, municipalities, local stakeholders and university). Overall, the design took about 9 months, from January to September 2019.

### Mobile healthy living room

**Fig. 2:**
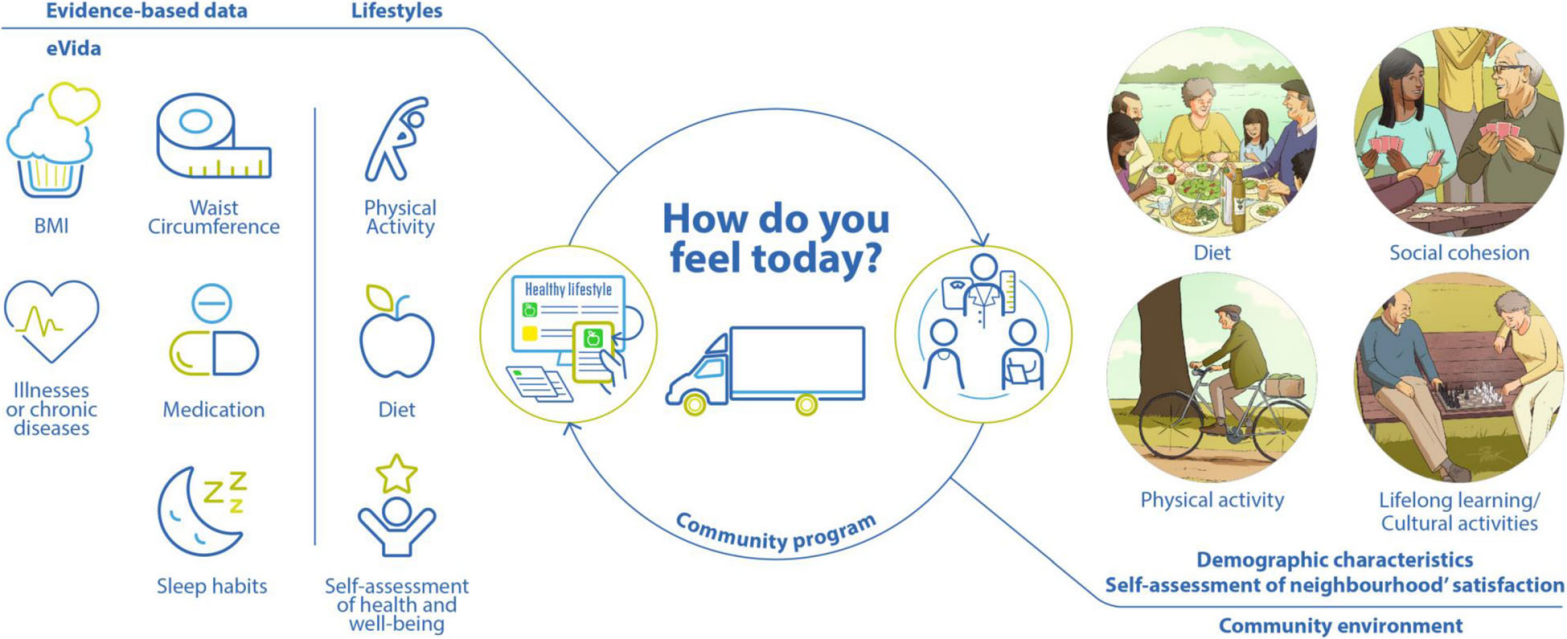
Community program intervention design; credits: the research team

The community program took place in a mobile Healthy Living Room (mHLR) (Fig. 2), designed as a mobile community service, to reach isolated rural neighbourhoods with lower access to health care facilities and awareness about healthy lifestyles. The mHLR was equipped with a healthy lifestyle assessment toolkit, which comprises medical devices and a questionnaire [27] incorporated in eVida technology. eVida is a tablet-based application centred on the input of the questionnaires (as discussed in detail below), provides a personalized summary of putative health risks associated with individual characteristics and behaviours [28].

The community intervention involved 1) the assessment of evidence-based data (e.g., BMI, waist circumference, and self-assessment of illnesses or chronic diseases, medication and sleep habits), 2) lifestyle characterization (e.g., diet, active lifestyles, quality of life and self-assessment of health and well-being), 3) demographic information (i.e., sex, age, employment status and level of education), complemented with 4) the self-assessment of neighbourhood satisfaction, all incorporated in eVida technology, and 5) the individual in-depth interview about the contexts in place to pursue good health in the neighbourhood. Each participant was accompanied by a trained team member and community intervention included two to four team members and four to six students/young professionals, depending on the neighbourhoods’ population.

At the end, participants received the results of the eVida questionnaire and prevention recommendations in an individualised report as well as short cartoon-like active healthy lifestyles messages, about diet, physical activity, social cohesion, and mental health and well-being.

This research was part of a collaborative European research project, Healthy Lifestyle Innovation Quarters for Cities and Citizens (HeaLIQs4Cities), funded by the European Institute of Innovation and Technology for Health (EIT Health), that unite researchers and neighbourhoods from Coimbra (Portugal), Groningen (The Netherlands) and Copenhagen (Denmark), around the concept of health capability and drivers of health and well-being. Among the stakeholders, the consortium Ageing@Coimbra represents a reference site in Centro region of Portugal within the European Innovation Partnership (EIP) on Active and Healthy Ageing (AHA), that is founded on a quadruple helix-based innovation model for improving active and healthy ageing in Europe [28].

### Data collection

One dimension of the data aimed at collecting evidence-based data, lifestyles and self-assessment of neighbourhood satisfaction incorporated in eVida, as mentioned above, while another dimension of the data aspired at documenting the contexts in place influencing the ability to pursue health and well-being in the neighbourhood. The weight and waist circumference were measured and BMI assessed; the factors associated with illnesses or chronic diseases, medication, and sleep habits were self-reported. The quality of life followed EQ-5D-5L questionnaire: mobility, self-care, usual activities, pain/discomfort and anxiety/depression (each dimension is rated on scale with 5 levels: no problems, slight problems, moderate problems, severe problems and extreme problems). We also considered two additional dimensions of self-assessment of health and well-being of ‘quality of life’ (with 5 levels, strongly disagree, disagree, neither agree nor disagree, agree, strongly agree) and ‘health condition’ (with 5 levels, very good, good, reasonable, bad, very bad). Regarding the description of lifestyles, diet was categorized per food groups per day and per week (following 5 levels in the Likert scale).

Qualitative research advances the possibilities of a deeper understanding of people’s perceptions and expectations and exploring unique topics within the research aims. For that purpose, we conducted the open-ended question in an in-depth interview: “*What would you change in your neighbourhood to have a healthier life?*”. To reduce eventual desirability bias, participants were ensured prior the eVida questionnaire that were no right or wrong responses and a privacy environment was ensured during the interview; the eVida and interview took in between 45 to 60 min.

Through eVida, information was collected on a random sample of 270 individuals living in rural neighbourhoods from the Sicó-network, considering the dimension and location of the neighbourhood (small villages, parish council and municipalities), constituting a sample with a margin of error of 5.75% and confidence level of 95%. The sample size for the interviews was determined by applying the saturation point criteria, and was stopped after 107 testimonials were collected. This study design was considered the most appropriate way to describe individuals’ lifestyles and communities’ environments. The collection of QUAL+quan data was performed by researchers with background on life sciences, medical and health sciences, and social sciences; the CBPR approach from the very early stages revealed to be determinant for the research methodology and outcomes. Furthermore, the first day of intervention was followed by a preliminary assessment and discussion by the advanced training students (and young professionals) and the team, in order to identify personal bias, optimize the use of eVida and the interview, and minimize any other form of unintended coercion with participants. Data collection was conducted between September 4 and 23, 2019.

### Ethical considerations

This study was approved by the Ethics Committee of the Centre Regional Health Administration of Portugal: Reference 91/2019. Participants were required to be 18 years or older and were asked to sign a written informed consent before initiating the community intervention. At the end, participants received a bag with the individualised report and the short cartoon-like active healthy lifestyles messages, about diet, physical activity, social cohesion, and mental health and well-being.

### Data analysis

Testimonies were documented in writing, and then transcribed and translated to English. Each participant was linked the age, sex and municipality council in order to present direct quotations (e.g., Female, 68, Small Village, Pombalinho 610). The first four authors performed an independent analysis in all testimonies developing a parallel codification on drivers of health and well-being at community level in the rural neighbourhoods. After several collective discussions rounds (over a period of 3 months), seven consensual dimensions were identified a priori: economic development, built environment, social network, health care, demography, active lifestyles and mobility. The a priori themes were used to code the qualitative data in which subtopics were built upon [29].

All testimonies were imported to MAXQDA Analytics Pro 2020 version 20.0.0 (Berlin, Germany: VERBI Software GmbH) for coding and analysis. The coding was done in three stages. In the first stage, the testimonies were coded based on the selected dimensions. In a second stage of coding, the resulting identification of sub-topics for each of the 7 dimensions based on mention frequency, and the identification of predominant topics, in both individual accounts and different neighbourhoods, was carried out independently across researchers. Any new codes were consensually debated during regular team meetings. In the third stage, all testimonies were coded once more by applying the final coding scheme. All coded testimonies were evaluated for emerging topics. We used several strategies to ensure quality in data coding. The composition of coding pairs was changed after 10 to 15 testimonies to reduce possible systematic bias. Using this approach, we were able to examine the in situ community needs in the 15 rural neighbourhoods. We also documented the clear individual positive perceptions of living in rural neighbourhoods: i) in terms of healthy living and well-being; ii) the different ways of describing and explaining lifestyles and daily habits; iii) the multiples ways of living and be engaged with community environment; iv) access to health care and health services.

Authors involved in the analyses maintained the explanatory map of the CBPR process from the research goals to data collection and analysis. The number and the frequency of subjects mentioned by participants in different topics support the reliability and credibility of our findings. We also used the lexical search on the MAXQDA program for key codes, to identify the frequency and number of mentions for consistency in participants’ responses.

To supplement the qualitative analysis, binomial logistic regression models were applied: BMI (classified in two categories: 1. overweight and obesity and 2. normal and low weight), waist circumference (classified in two categories: 1. and 2.), self-assessed health status (classified in two categories: 1. good and very good and 2. less than good), were assessed as dependent variables and sex, age (continuous), place of residence (classified in the three classes: 1. small villages, 2. parish councils and 3. municipalities), as independent.

## Results

### Demographic characteristics

Two hundred seventy people participated (84 in small villages, 112 in parish councils and 74 in municipalities). Women made up a larger proportion of the participants (63%) in the three levels (Table 1). The median age was 69 years (1st quartile: 58 years; 3rd quartile: 77 years), with 78% of the participants above 55 years of age. Most of the participants were retired (64%), with a higher proportion (77%) in small villages. The level of education varied along the neighbourhoods, with a small proportion (9%) having receiving higher education (4 and 3% in small villages and parish councils, and 24% in municipalities); the largest share of participants completed the first two grades of basic education (69%), and 14% did not receiving primary education (29, 9 and 7% in small villages, parish councils and municipalities, respectively).

### Individual health functionality

**Fig. 3:**
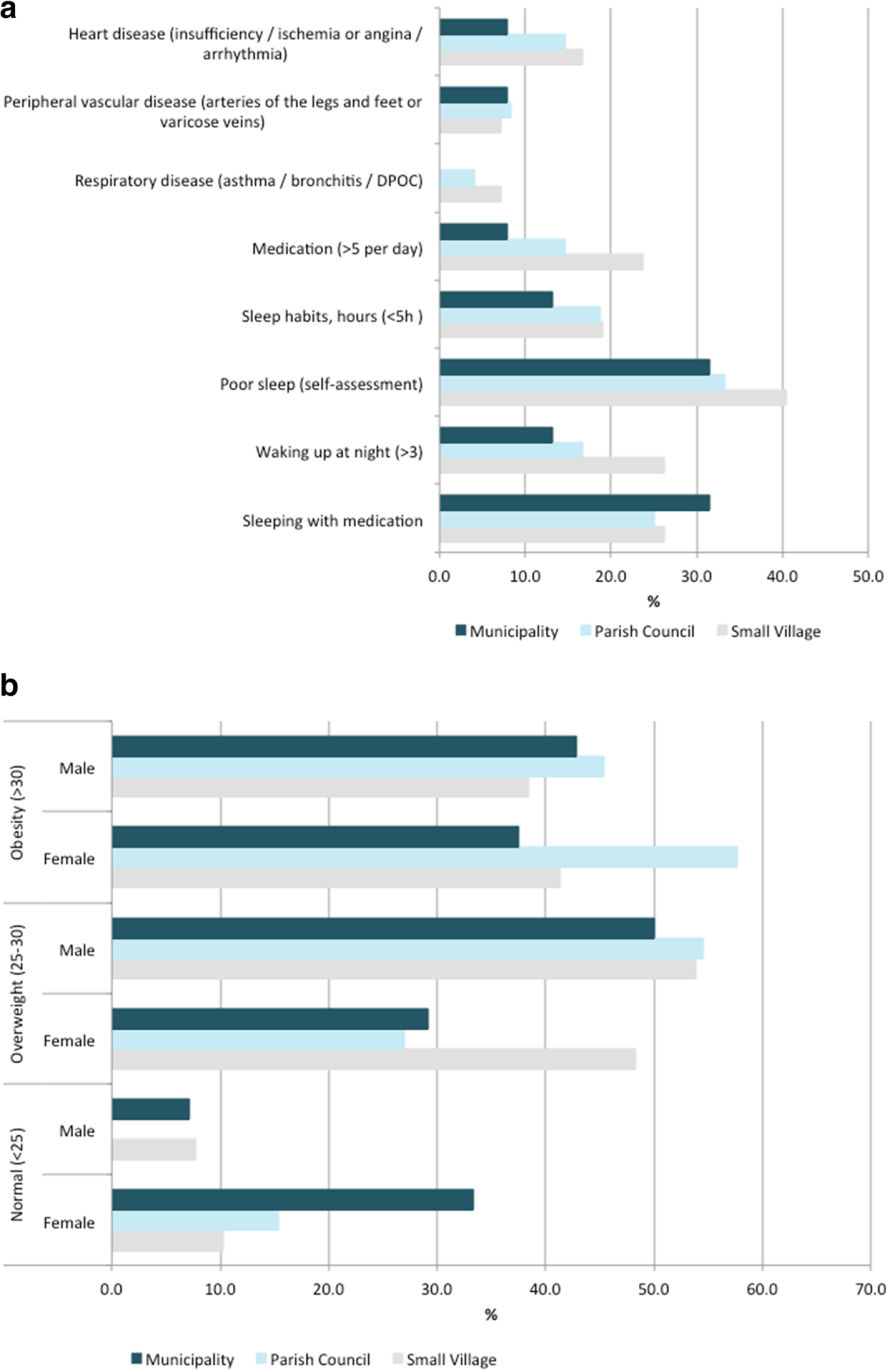
Evidence-based data by rural neighbourhood for participants aged 55 to 74 years

The proportion of participants with normal BMI was substantially lower above 55 years of age, with a higher proportion of women presenting normal BMI than men for the participants aged 55 to 74 years (Additional file 1: Table S1). The proportion of participants with obesity was slightly higher in women aged 55 to 74 years and lower in the other range of ages (< 54 years, > 75 years). In terms of obesity data by rural neighbourhood, the proportion of participants with obesity was lower in municipalities (32%) than in small villages (38%) and parish councils (45%). For the participants aged 55 to 74 years (Fig. 3a), excess weight was lower in women in all types of neighbourhoods (48 and 54% in small village; 27 and 55% in parish councils; 29 and 50% in municipalities; respectively); obesity was higher in men in municipalities (43 and 38%, respectively). Overall, men had a 75% higher probability of being overweight than women (*p*-value: 0.0954), while waist circumference measurements reflected obesity over age, being consistently higher in participants > 75 years of age; the risk of having high waist circumference was 2.45 (IC: 1.1; 5.7) times higher in individuals living in small villages than in municipalities.

NCD risks associated to chronic diseases were reported by 25% of the participants aged 55 to 74 years (Fig. 3b) including: (i) heart disease (heart failure, ischemia or angina, arrhythmia) was declared by 13% (17% in small villages, 15% in parish councils and 8% in municipalities); (ii) peripheral vascular disease (problems in arteries of the legs and feet, or varicose veins) was mentioned by 8% (7% in small villages, 8% in parish councils and 8% in municipalities); and (iii) respiratory disease (asthma, bronchitis, chronic obstructive pulmonary disease) was declared by 4% (7% in small villages and 4% in parish councils) (the information for all participants is presented in supplementary Table S1). The lowest prevalence of medication was documented in parish councils (33%) and municipalities (32%) (Additional file 1: Table S1); overall, a substantial proportion of the participants (59%) reported were taking 2–5 medications a day, and a lower proportion (16%) reported taking > 5 medications a day.

Sleeping habits ranged from ≥7 h for 38% of the individuals and less than 5 hours for 17% of the individuals, with a clear trend of more sleeping hours in individuals living in municipalities (Additional file 1: Table S1). Sleep without interruption was reported by 48% of the individuals, with higher prevalence (55%) in individuals living in municipalities. Consistently with sleeping hours, 35% of the individuals considered having poor sleep quality (41, 33 and 32%, in small villages, parish councils and municipalities, respectively; supplementary Table S1).

Self-rated health condition ranged from good (47%) to reasonable (42%), with little differences in neighbourhoods. About 5% of participants referred having very good health, consistently in all neighbourhoods, contrasting with the 5% of participants that mentioned having bad health, with lower incidence in municipalities (3%). Severe or extreme pain was reported by 6 and 1% of the individuals, respectively, with higher incidence from participants living in small villages. In terms of self-rated well-being, a large proportion of participants (74%) reported having a good quality of life, with 25% of the individuals attributing the highest score (18% living in small villages, 30% in parish council and 26% in municipalities). Across data, participants with higher waist circumference had a 2.21 (IC: 1.19; 4.27) higher probability of presenting a poor self-evaluation of their health status.

### Unhealthy lifestyles according to rural neighbourhood type

The description of lifestyles in the 15 rural neighbourhoods is shown in Table 2. A large proportion of participants (81%) reported eating fruit and vegetables 0–1 times per day. Only 1% of the participants mentioned eating fruit and vegetables fewer than once. A substantial proportion of individuals reported eating fish, meat and eggs (87%) 0–1 times per week in all neighbourhoods; also, a considerable share of individuals reported eating bread, pasta or cereal (82%) 0–1 per day, ranging from 78% in parish councils to 89% in small villages. Many participants reported drinking milk (66%) 0–1 per day, ranging from 50% of individuals living in municipalities to 60% of respondents from small villages; 6 and 9% mentioned drinking milk once a week or never, respectively, with little differences in all neighbourhood types. The majority of the population (69%) referred eating fried and salty foods once a week or less, in all neighbourhoods. Some participants (59%) mentioned eating sweets once a week or never, and 7% reported eating more than once a day (2% in small villages, 11% in parish council and 8% in municipalities). Regarding active lifestyles, a large proportion of participants (67%) reported having daily active routines. A lower proportion of participants (21%) reported regular vigorous physical activity, ranging from 11% doing gymnastics (e.g., fitness, Pilates, yoga), 4% water-based exercise (e.g., swimming or water aerobics), 2% bicycling, 1% running and 3% other sports. In general, those living in the municipalities assess better quality of life (following EQ-5D-5L questionnaire); regarding the self-assessment of health and well-being, the inferior levels were observed in small villages.

**Table 2:** Individual lifestyles by neighbourhoods’ type

Diet, n° times per week	Small village	Parish Council	Municipality	Total
	(*n* = 84)	(*n* = 112)	(*n* = 74)	(*n* = 270)
Vegetables and fruit				
Never	–	–	1 (1%)	1 (0.4%)
< 1	–	1 (1%)	1 (1%)	2 (1%)
1–3	3 (4%)	7 (6%)	3 (4%)	13 (5%)
4–6	14 (17%)	11 (10%)	9 (12%)	34 (13%)
Once or more per day	67 (80%)	93 (83%)	60 (81%)	220 (82%)
Milk and dairy products				
Never	8 (10%)	11 (10%)	6 (8%)	25 (9%)
< 1	4 (5%)	8 (7%)	5 (7%)	17 (6%)
1–3	7 (8%)	12 (11%)	8 (11%)	27 (10%)
4–6	7 (8%)	5 (5%)	11 (15%)	23 (9%)
Once or more per day	58 (69%)	76 (68%)	44 (60%)	178 (66%)
Fish, meat and eggs				
Never	–	–	–	–
< 1	–	1 (1%)	–	1 (0.4%)
1–3	1 (1%)	4 (4%)	5 (7%)	10 (4%)
4–6	11 (13%)	9 (8%)	4 (5%)	24 (9%)
Once or more per day	72 (86%)	98 (88%)	65 (88%)	235 (87%)
Bread, pasta or cereal				
Never	–	–	–	–
< 1	1 (1%)	–	1 (1%)	2 (1%)
1–3	4 (5%)	8 (7%)	6 (8%)	18 (7%)
4–6	4 (5%)	17 (15%)	8 (11%)	29 (11%)
Once or more per day	75 (89%)	87 (78%)	59 (80%)	221 (82%)
Legumes and grains				
Never	2 (2%)	–	3 (4%)	5 (2%)
< 1	4 (5%)	11 (10%)	6 (8%)	21 (8%)
1–3	27 (32%)	59 (53%)	28 (38%)	114 (42%)
4–6	31 (37%)	25 (22%)	28 (38%)	84 (31%)
Once or more per day	20 (24%)	17 (15%)	9 (12%)	46 (17%)
Fried and salty foods				
Never	13 (16%)	14 (13%)	11 (15%)	38 (14%)
< 1	46 (55%)	65 (58%)	38 (51%)	149 (55%)
1–3	23 (27%)	24 (21%)	20 (27%)	67 (25%)
4–6	2 (2%)	7 (6%)	3 (4%)	12 (4%)
Once or more per day	–	2 (2%)	2 (3%)	4 (2%)
Sweets				
Never	15 (18%)	19 (17%)	6 (8%)	40 (15%)
< 1	39 (46%)	44 (39%)	35 (47%)	118 (44%)
1–3	17 (20%)	21 (19%)	20 (27%)	58 (22%)
4–6	11 (13%)	16 (14%)	7 (10%)	34 (13%)
Once or more per day	2 (2%)	12 (11%)	6 (8%)	20 (7%)
Active lifestyles				
Regular activity	27 (32%)	49 (44%)	31 (42%)	107 (40%)
Walking	17 (20%)	35 (31%)	22 (30%)	74 (27%)
Cycling	1 (1%)	4 (4%)	–	5 (2%)
Collective games	1 (1%)	–	3 (4%)	4 (2%)
Outdoor running	1 (1%)	–	–	1 (0.4%)
Holistic movement practices: yoga, Pilates	5 (6%)	13 (12%)	9 (12%)	27 (10%)
Fitness equipment	1 (1%)	1 (1%)	2 (3%)	4 (2%)
Water-based exercise	1 (1%)	8 (7%)	2 (3%)	11 (4%)
Other(s)	2 (2%)	4 (4%)	1 (1%)	7 (3%)
Quality of life^a^				
Mobility				
No problems	52 (62%)	74 (66%)	54 (73%)	180 (67%)
Slight	19 (23%)	16 (14%)	9 (12%)	44 (16%)
Moderate	9 (11%)	18 (16%)	10 (14%)	37 (14%)
Severe	4 (5%)	4 (4%)	1 (1%)	9 (3%)
Extreme	–	–	–	–
Self-care (washing or dressing)				
No problems	74 (89%)	106 (96%)	66 (89%)	246 (92%)
Slight	4 (5%)	4 (4%)	7 (10%)	15 (6%)
Moderate	2 (2%)	1 (1%)	–	3 (1%)
Severe	–	–	–	–
Unable to self-care	3 (4%)	–	1 (1%)	4 (2%)
Usual activities				
No problems	67 (80%)	91 (82%)	57 (77%)	215 (80%)
Slight	10 (12%)	18 (16%)	13 (18%)	41 (15%)
Moderate	5 (6%)	1 (1%)	3 (4%)	9 (3%)
Severe	–	–	–	–
Unable to do usual activities	2 (2%)	1 (1%)	1 (1%)	4 (2%)
Pain/discomfort				
No problems	15 (18%)	38 (34%)	32 (43%)	85 (32%)
Slight	42 (50%)	42 (38%)	29 (39%)	113 (42%)
Moderate	19 (23%)	24 (21%)	10 (14%)	53 (20%)
Severe	7 (8%)	7 (6%)	3 (4%)	17 (6%)
Extreme	1 (1%)	–	–	1 (0.4%)
Anxious or depressed				
No problems	40 (48%)	50 (45%)	42 (57%)	132 (49%)
Slight	30 (36%)	39 (35%)	18 (24%)	87 (32%)
Moderate	9 (11%)	18 (16%)	7 (10%)	34 (13%)
Severe	5 (6%)	5 (5%)	7 (10%)	17 (6%)
Extreme	–	–	–	–
Self-assessment of health and well-being				
Quality of life				
Strongly disagree	2 (2%)	2 (2%)	1 (1%)	5 (2%)
Disagree	7 (8%)	9 (8%)	4 (5%)	20 (7%)
Neither agree nor disagree	18 (21%)	13 (12%)	13 (18%)	44 (16%)
Agree	42 (50%)	54 (48%)	37 (50%)	133 (49%)
Strongly agree	15 (18%)	34 (30%)	19 (26%)	68 (25%)
Health condition				
Very Good	3 (4%)	6 (5%)	4 (5%)	13 (5%)
Good	43 (51%)	46 (41%)	38 (51%)	127 (47%)
Reasonable	32 (38%)	53 (47%)	29 (39%)	114 (42%)
Bad	6 (7%)	5 (5%)	2 (3%)	13 (5%)
Very bad	–	2 (2%)	1 (1%)	3 (1%)

^a^Following EQ-5D-5L. Data are in %, some percentages might not add to 100% due to the option given to subjects of not answering

### Characterization of community environment

**Fig. 4:**
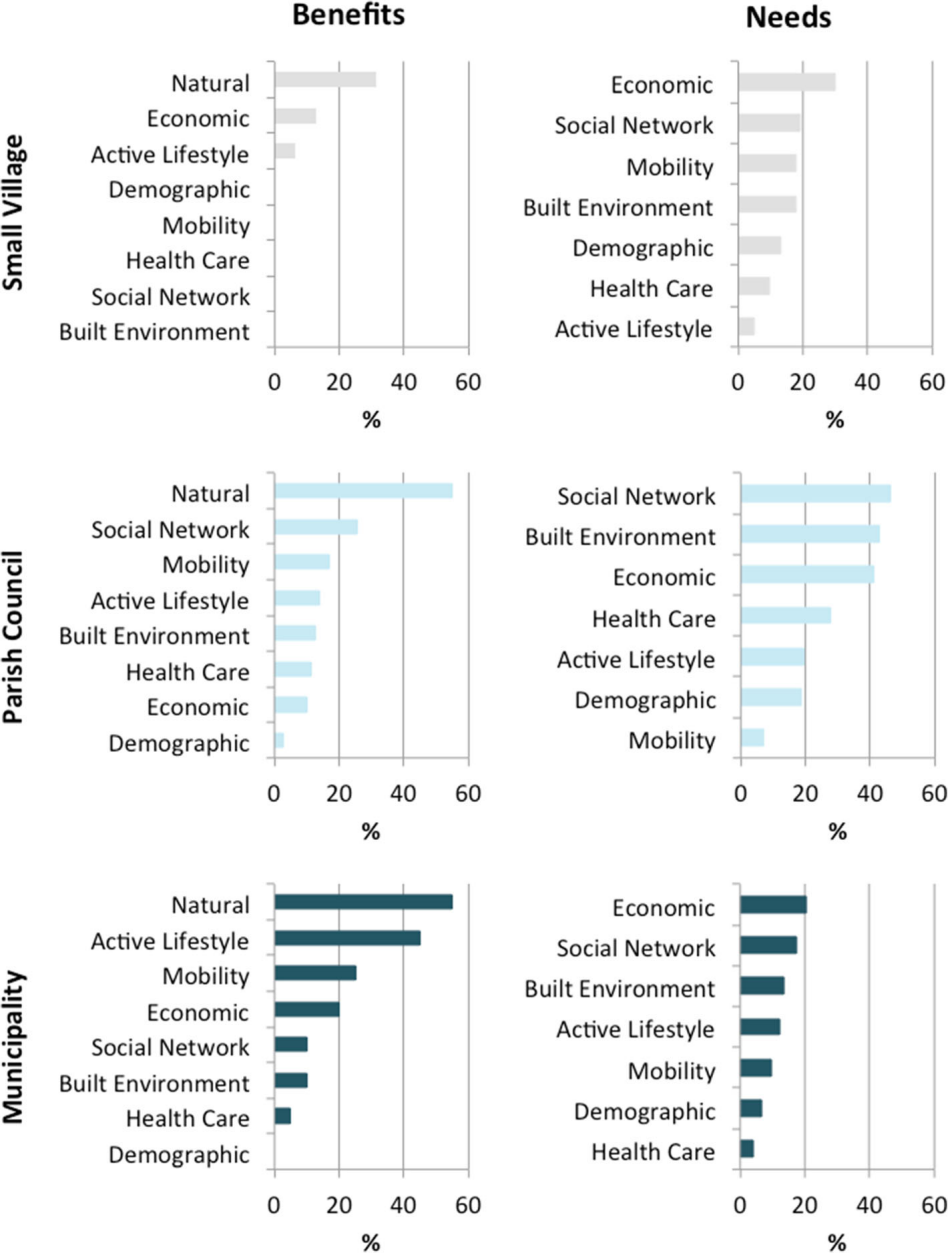
Individual’s reflections about community circumstances influencing health and well-being in their rural neighbourhood

Individual reflections pinpointed seven dimensions as the main drivers to pursue health and well-being in rural neighbourhoods. These include: economic development, built (and natural) environment, social network, health care, demography, active lifestyles and mobility (Fig. 4; supplementary Table S2). Such reflections envision people-centred expectations and a deeper understanding of valuable surrounding environments connected to well-being, which contribute to unforeseen wider ‘needs’ and ‘benefits’ of rural areas.

One third of the participants (86) stressed economic development as the main community need –financial, technological and digitalisation investment, high-value-added industry, industrial infrastructures, digitalisation for remote working–, with particular focus on economic innovation and diversification to encourage the establishment of young people in rural areas. Regional policies to improve investment and attractiveness of high-skilled young workers were mentioned by 12 participants.

Built environment, goods and services, underlined by 73 participants, emphasize the need for maintenance and conservation of (i) infrastructures for social interaction, ranging from cultural activities (24), green-blue areas for practicing physical activity and exercise, e.g., green public spaces, camping areas, river beaches, playing areas for children (18), to connected green-blue infrastructures for enjoying nature (14); (ii) infrastructures for promoting the inclusive walkability, namely for youth and elderly people with morbidities, such as smooth and safe walking paths and resting places (10) or sound barriers (2); and (iii) the patrimonial rehabilitation for tourism and habitation (3). Among the services needed, cafes, grocery stores or restaurants, bank, book stores and shopping facilities were mentioned. However, built environment reflected asymmetries in the neighbourhoods; some participants (7) underlined the accessibility to cafes, supermarkets and restaurants in their respective neighbourhoods as an additional benefit of living in rural areas, while others (11) mentioned safe streets, infrastructures for practicing exercise, e.g., gymnasium, swimming pool, tennis court and walking routes, and cultural activities, e.g., folk activities, folk music, cinema and theatre.

Social relationships and networks in neighbourhoods, mentioned by 81 participants, included local community-based initiatives and means of communication to reinforce social connections and dynamics. Asymmetrically, other participants (20) reinforcing local networks and dynamics as a benefit of living in their neighbourhoods, exemplifying with the active participation in collective grape/olive picking, or cultural and recreation activities.

Health care, mentioned by 42 participants, was mostly associated to elderly dependency and included the need for better and long-term health care services (39), support in transport to health care services (1) and pharmacies (2). Asymmetrically, the suitable health care support and services, primary health care services and pharmacies, emphasized by 9 participants, reflected the beneficial aspects mentioned in some neighbourhoods. Adult social care support, particularly day centres and nursing homes, underlined by 21 participants, including childcare and family care were also among the needs reported in rural neighbourhoods.

Demographic factors, mentioned by 37 participants, focused particularly on population ageing and the need of (young) people (30) as social pressure to improve education and (re)open schools (3) and kindergartens (3). The local education, stressed by 2 participants, was reported as a main benefit in their own neighbourhood, to promote well-being.

Active lifestyles, emphasized by 35 participants, include the need for (i) lifelong learning opportunities and digital inclusion, e.g., internet, information and communication technologies (ICTs) (17 participants); (ii) access to places for practicing physical activity and exercise, e.g., soccer, yoga, Pilates, fitness, pool, and walking (10 participants); and (iii) cultural activities, e.g., dance, music, cinema (8 participants). Mobility, mentioned by 30 participants, included the need of accessible public transport (25) and safe accessible walking routes (5). Asymetrycally, several participants (16) underlined the functionality in mobility –public transports systems– and accessibility and linkages (highways) to villages and cities nearby as a main benefit of their neighbourhood.

Natural resources and natural environment were in the centre of health and well-being in rural neighbourhoods. The majority of the individuals (237) mentioned to like living in their neighbourhood and 55 participants featured the natural environment was as the main community benefit to improve quality of life, describing their neighbourhood as calm, beautiful, healthy and safe. The prioritisation on quality of life include (i) daily routines linked to land use, e.g., gardening, agriculture, silvo-pastoral practices; (ii) biodiversity; (iii) connectivity with nature, e.g., swimming and fishing in rivers, walking in green spaces, woodlands and mountains; and (iv) environmental quality, e.g., lower exposure to air / noise pollution. More than two thirds of the participants (192) mentioned they would not live elsewhere if they could and one third (92) revealed they would not change anything in their neighbourhood. Overall, 216 participants underlined that their own neighbourhood is a good place to live. Specific testimonies on these issues are sampled below (Table 3).

**Table 3:** Individual’s testimonies

**Economic development**	
“*It’s a good neighbourhood to live but lacks jobs! My son had to move to a city. There is no industry to employ young people. It is a good place to live, anyway. It’s a good place to grow old… has nursing homes.*” Male, 55–60, Parish Council (Espinhal 583) “*There is a lack of jobs, we need more industry to attract young people. We have an industrial area, but is not enough; it has few companies and pay poorly.*” Male, 60–65, Parish Council (Espinhal 581) “*It’s a quiet neighbourhood, but it could have better living conditions; the population is very old. There should be more investment to attract (young) people, because it is good to live here. We are close to cities.*” Female, 60–65, Parish Council (Redinha 537) “*We are from here. I was away (in a city) for some time, five years, almost, to do an extension of my studies. Then, with family, I decided to come and to stay (...) It is needed to work on attracting more investment to empower the neighbourhood at all levels. (...) With more investment, the socio-economic (development) will pull the remaining sectors. I am an electrical engineer, I used to work on innovation in another city; now I work in a city nearby, I develop software. There is an increasing flexibility (about working from home) in our area. No, I don’t earn more (money). (...) Life is not just money. Her childhood is worth than a few euros more! (...) By working from home, I can go further (professionally) and be with my daughter.*” Male, 30–35, Municipality Council (Penela 490) “*I wouldn’t change anything. (…) It’s quiet. Lately, many people are buying houses to restore; there is more investment. (laughs) I like it very much.*” Female, 70–75, Parish Council (Zambujal 507)	
**Built environment**	
“*I have a coffee shop. The business is going well, but it has gone better in the past. We used to have a school; classes started to decrease in number of kids and the school closed. They (kids) used to came here to eat and drink; not anymore.*” Female, 75–80, Parish Council (Redinha 704) “*I really enjoy living here. (…) We need a supermarket, a nursing home, a kindergarten; these are the most necessary things. (…) I used to live and work in a city, but I didn’t like it. It’s calmer here. I like it!*” Female, 50–55, Parish Council (Zambujal 526) “*It’s a good neighbourhood to live. We have a bank, a pharmacy, a supermarket and a coffee shop. However, activities for family are lacking for example. There is no place to take children to play, not even a playground.*” Female, 40–45, Parish Council (Redinha 539) “*We need a mini market and coffee shop to socialize. I have no driving license; the bus comes on Tuesdays and Fridays and takes us to the market. (…) People leave to places with more services.*” Female, 55–60, Small Village (Serra de Janeanes 547) “*What would change (laugh)… playground for children! Areas for doing sports. Leisure spaces. Hiking routes, (the walks) they are done on the side of the road. Gymnasium. (…) (On the other hand) We have it all! We have mountains. We have water. We have the river. We have (rural tourism) people who come from France, Spain, from all over the country, to hiking. (…) We have a health center, a large market, a pharmacy, a post office, schools. We live without pollution. (…) This small village has a lot to offer, but many people leave because of bureaucracy. (…) People from my generation left (the neighbourhood). When I was young, this square (central square of the neighbourhood) had always children playing and the river full of children and adults. (…) The river used to be cleaned every year. Now it’s not allowed to clean, to protect some plants, a water lily, the fish ... But now it is covered with reeds, branches of trees, and you can hardly see it. (…) We have everything and we have nothing! We have a folk group. (…) Maybe I am aware because I work at a school. (…) A kindergarten is needed (…) There are no natural pools. (…) We cannot enjoy because there are no (safety) conditions. (…) We have many foreign tourists visiting us, some sleep in their cars... there are no infrastructures*” Female, 45–50, Parish Council (Redinha 557) “*The neighbourhood has great offerings, personal trainer, yoga, gym, swimming pools, walking green spaces. This is great. It’s amazing! (Local government) stimulate cultural activities. We see people willing to come back. I went to the capital 10 years ago (for studying); I still go once or twice a week, I have my office there. I work in the financial area. We need to change our mindset and working from another place, outside the conventional office.*” Female, 30–35, Municipality Council (Penela 487) “*I wouldn’t change anything. Because what I really need is to change my lifestyle. Here (neighbourhood), there is a little bit of everything. I think it (neighbourhood) has good initiatives, culture and services. (…) We are close to everything. I would live somewhere else. Maybe. If I moved, maybe I would enjoy it, I have quit a lot work here*.” Female, 20–30, Municipality Council (Penela 498)	
**Social relationships and networks**	
“*I would like more proximity between neighbours. I go out and I don’t see anyone! We used to have a place for socializing, but not anymore. I wish there could be a place for people being together, to meet and do activities. Nowadays, we have to get used to loneliness.*” Female, 80–85, Parish Council (Alvorge 717) “*We do have a health care center, a nursery house, an elderly center. Some people have reduced mobility to move, some neighbourhoods have a van to transport people to the health care center. I like to live here, (but), if I could, I would live somewhere else. I do miss being with people.*” Female, 65–70, Parish Council (Zambujal 524) “*There is a need for social networking or activities for socializing. (…) Another good idea would be exercising equipment installed near the river. (...) Tourism. There is grape harvesting (rural tourism) in some neighbourhoods; here it could be olive picking and harvesting. Our olive oil is really good, and many olive trees are not harvested.*” Female, 50–55, Parish Council (Redinha 536) “*We need a nursing home, so that our neighbours do not need to leave (...) I really like living here, people are friendly with each other. It’s spectacular! We are in grape harvest every day, now. Everyone helps each other. (pause) Some things are missing, someone to teach us those (information and communication) technologies. We have a youth association. We had a football team, but not anymore.*” Female, 65–70, Parish Council (Zambujal 509) “*It is a quiet neighbourhood (to live), but people are too closed. It would be nice to have a place for us to meet and socialize (pause). They (local representatives) made an infrastructure for social activities (in the village) but it’s very cold, even in the Summer. Before, there was a school, right here, but they did something else*.” Female, 70–75, Parish Council (Alvorge 713) “*It’s a very peaceful place. Yesterday, in the grape harvest, we were about fifty people. Neighbours, we help each other*.” Female, 65–70, Parish Council (Zambujal 519) “*We have olive oil, wine, chickpeas, beans, grapes, figs. We have grape harvesting. This year didn’t go so well; there is less grape production. We will have good olive oil; if everything goes well, there is a lot of olives (in the trees). We have good olive oil. (...) Tomorrow I will have many people harvesting for me. I’ve fried small sardines (“petinga”) and I made bread potato (dessert). (laughs) There is a competition for the best tasty tidbit (laughs)*.” Female, 55–60, Small Village (Serra de Janeanes 547) “*I wouldn’t change anything. I am satisfied with everything. I used to live in big cities and here it’s better for my child. My children can walk freely on the street. Neighbours know each other and everything is fine.*” Female, 45–50, Parish Council (Alvorge 784)	
**Health care support and services**	
“*A doctor; there used to be. A nursing home; there used to be. (…) About 200 people are living here. But a lot of people used to live here. (…) A half dozen of new couples live here, now. (...) We don’t have anything here. We don’t have a nursing home, we don’t have a supermarket, we don’t have a doctor. (…) I would move to a place with everything.*” Female, 70–75, Parish Council (Zambujal 502) “*Health care center (in the neighbourhood) is not working well, we need to wait 2–3 months for a medical appointment.*” Male, 70–75, Parish Council (Espinhal: Santa Eufemia 572) “*We have everything! It is a great neighbourhood to grow old; we have 2 nursing homes and 3 private elderly health care centers. (…) It’s very expensive.*” Male, 60–65, Parish Council (Espinhal 581) “*The health care center is good, except when the doctor is on vacation.*” Male, 80–84, Parish Council (Alvorge 714)	
**Demography**	
“*I live in a small village, four kilometers (from the neighbourhood). There are three houses and seven people: four of my age, two children (mine) and one older woman (mother). I am a social worker. Ten years in the field doing work, the number of people is decreasing. During the day, my mother is the only person at the village.*” Female, 45–50, Municipality Council (Penela, working place, 489)	
**Mobility**	
“*More public transport to the city, to schools, and to all residents and their schedules. Schools have about 300 students (from different neighbourhoods). There are three buses to the city, which forces us to ride a car. I’m a music therapist and I work in more than one place.*” Female, 20–25, Municipality Council (Penela 479)	
**Natural environment**	
“*It is a nice neighbourhood, quiet; I wouldn’t change for anything else.*” Male, 80–85, Parish Council (Podentes) “*I love to live here. It is a very healthy neighbourhood.*” Male, 55–60, Parish Council (Espinhal) “*I have a garden with everything. I take care of the garden. We eat from the garden.*” Female, 65–70, Small Village (Pombalinho 610) “*Up there (in the mountains) there is a small church, in days with good visibility, not today, one can see the sea. We have beautiful landscapes!*” Female, 45–50, Parish Council (Redinha 557) “*I am from a city near the capital and I my whole family lives there. (...) My husband is from here. I like it here, more. This landscape. This quietness. This calmness. This fresh air. This beauty (landscape). We have everything. What would we need after getting old? Nothing, just calmness. (laugh) (…) There is a lack of social activities, some goods, a shop with books for example, I really like to read.*” Female, 75–80, Parish Council (Espinhal 571) “*It is a very beautiful neighbourhood. It has a lot of water, I really like to live here.*” Male, 80–85, Parish Council (Espinhal: Santa Eufemia 755)	

## Discussion

To the best of our knowledge, this is the first qualitatively driven mixed-method approach to assess whether unhealthy lifestyles and surrounding environments are reflected into metabolic risks and health capability at individual and community-level in rural neighbourhoods.

In terms of the main findings, excess weight and obesity are more prevalent in men between 55 to 74 years and in individuals younger than 54 years, respectively, while in women obesity predominates between 55 to 74 years while excess weight is more predominant in individuals younger than 54. Considering the overall population, NCD risk linked to BMI was superior in small villages than in municipalities. NCD risk associated to unhealthy lifestyles was less evident for diet and sleep habits than for (lack of) physical activity. Diet habits reported by the participants strongly evidenced the adherence to a Mediterranean dietary pattern, which is linked to healthy lifestyles due to its protective effect against several metabolic risks and NCD, namely type 2 diabetes mellitus (T2DM), cardiovascular disease (CVD), obesity, cancers and total mortality [30]. Diet and metabolic risks were described as the second and third leading risks factors of early mortality in a recent survey for Portugal [31]; but it did not address rural and urban neighbourhoods separately. In Europe, DALYs and risks evidences from NCD also often expose dietary and metabolic risk factors [12], but again little is known about the relationship between NCD burden and community environment. Healthy diet habits reported in our study suggest that the accessibility to healthy food in own gardens and farms as well as in local markets enable the ability to make healthy choices. Indeed, several participants from small villages mentioned they produce their own food (e.g., vegetables and legumes, fruits and nuts, cereals, meat, eggs, cheese, olive oil), whereas participants from parish councils and municipalities mentioned obtaining local products in grocery stores or the local weekly markets.

Low level of regular physical activity and exercise was admitted by most of the participants in all neighbourhoods. Physical inactivity has been recognized as the fourth leading risk factor for global mortality [32] and the most pressing public health burden of the current century [33]. Portugal is the second country in the euro-area with higher physical inactivity in people over 60 years of age and among the countries with higher prevalence of multi-morbidity in people between 60 and 65 years [34]. Two previous reviews have highlighted that physical inactivity may be explained by pursuing health focused on individual-level determinants, such as self-motivation or literacy, whereas surrounding environment also determines the ability to prevent metabolic risks and choose healthy lifestyles [35, 36].

The qualitative research revealed people-centred health and well-being expectations, allowing us to identify seven main dimensions in community circumstances: economic development, built and natural environment, social network, health care, demography, active lifestyles, and mobility, affecting the options to improve or pursue healthier lifestyles, with asymmetries among the neighbourhoods. In fact, participants reframed the narratives, “*I like where I am!*”, underlining the benefits of living in their own neighbourhood; while two thirds of the participants revealed they wouldn’t live elsewhere if they could. Several studies have previously researched the effect of place of residence in terms of availability and accessibility in order to improve health [37–40].

Economic development and built environment emerged as the main community needs, namely via financial, technological, and digitalisation investment to attract high-skilled young workers to rural areas, and social interaction and lifelong learning activities, respectively, given that built and natural environment are the setting for the development of human activities [41]. Natural resources and natural environment were stressed as the main value of rural well-being. However some participants mentioned missing planned and oriented structures to connect with nature, such as functional green-blue areas to exercise / be physically active, or socialize, which can be also an opportunity to come with co-benefits for biodiversity and nature protection and conservation [42]. Some rural neighbourhoods have been associated with less vigorous physical activity due to socio-economic disadvantages, including less availability to, and use of, facilities for sports and recreational activities [43, 44]. By contrast rural neighbourhoods with available green spaces and higher accessibility or walkability tend to contribute to metabolic risk prevention, namely for T2DM [36]. However the (perceived) accessibility of walkability in rural and urban neighbourhoods may vary in different parts of the world. The low use of the bicycle as a mode of transportation reported in our study can be associated with the absence of specific infrastructures for cycling safety (e.g. on-road bike routes, on-road marked bike lanes), mentioned by some participants, but could also be due to the (high) average participant age. Notably, previous qualitative studies have stressed the positive association between adapted designing interventions in the environment for promoting active lifestyles and PA in rural adults, with gains to social cohesion and individual health conditions [43–47].

Rural neighbourhoods in Portugal are characterized by a higher ageing index, lower geographical access to health care, lower average income and declining population [48, 49], but there still is an underestimation of health capability versus disease burden and environment. DALYs have been relevant in terms of the costs to direct health care, namely to the public sector [50]; however, the translation of such knowledge rarely results into positive contributions and policies to rural neighbourhoods [20]. Some key subjects need to be considered in further research, including whether the 1) prevalence of women is associated with the demographic uneven structure of the elderly populations, or with women involvement in community, such as agriculture and social activities; 2) increase in evidence-based health and well-being is accompanied by an improvement in community environment, and whether common causes of choosing to live in rural neighbourhoods, such as greater food security, safety, connection with nature, quality of environment, improve metabolic risks and NCD over time [8] and thus health capability. The ambition of creating accessibility of ‘health-promoting environments’ in green and public areas, to reduce the NCD is well reflected on goal 11.7 of the World Health Organization’s sustainable development goals (2016) [51]. Populations in rural areas have access to, among other things, healthy food and healthy environmental resources; however rural structural capacities are often under-represented in developing and implementing socioeconomic policies.

In fact, rural marginalization affects health and social justice [52] and impacts metabolic risks and co-morbidities in populations [5]. BMI and waist measures observed in this study combined with the participatory approach about lifestyles and community environment, configure an opportunity to act differently in terms of improving health capability in Portuguese rural neighbourhoods, and these findings could thus serve as a driving force for encouraging healthy changes at both individual and community levels [18].

There are some limitations to this study. The approach was conducted in a single region of the country; thus, results cannot be generalized to other rural neighbourhoods or remote regions. Moreover, data was collected during standard working hours of the week, which might have influenced the sample, including ageing index and the prevalence of women participating. However, we did cover a representative sample of rural populations in Portugal. The eVida has been designed to be user friendly and of almost immediate understanding to participants (10 to 20 min to complete) [27]. Although the eVida has been re-designed to record information about external environment factors, testimonies were mostly documented in writing and then transcribed. Future research in health innovation devices should also focus on developing programs that can incorporate context-based information, and with it, a better understanding of how ability to pursue health come as a whole from internal and external factors. The use of technology-based devices is increasingly modifying resources and support of health care services and health monitoring, traditionally carried out by health providers in medical facilities. Such innovative devices and adapted strategies have been suggested to encourage active self-management and to ‘empower’ behaviours, and as a way to acquire reliable health-related knowledge to make self-balance decisions [28].

The qualitative driven mixed-method design allowed us to gather data concerning unhealthy lifestyles of individuals but also to collect in-depth information about community environments that facilitate / weaken individual health and well-being, and their ability to make healthy choices (data saturation was achieved by characterizing broader determinants of health and well-being in neighbourhoods). We believe that the mixed-method described is one way to combine multiple components acting independently and inter-dependently, in order to better understand health capability at both the individual and community levels. The main strengths of the study include the co-designing community program involving the local representatives of the Sicó-network and advanced training students (and young professionals), working together with a trans-disciplinary research team. With the advantages of CBPR, the involvement of community in the early stage of the study provided the opportunity for discussing and adapting the health-related messages for a population with a high ageing index and limited literacy living in the Sicó-network (Portuguese National Statistics, 2019). Such involvement of community and its degrees of negotiation, and flexibility, enabled researchers to uncover gaps regarding (natural) environment contextual-dependent circumstances influencing the ability of individuals to pursue health in their own neighbourhoods.

Our findings are relevant for raising healthy lifestyles awareness and health seeking-skills to improve the self-ability to make balanced decisions, for implementing technology-based devices combined with participatory dynamics, as well as for encouraging the active engagement of local representative planners (governments and other stakeholders) in research to enhance the capacity building and thus the capability for improving heath in rural areas. There are specific contexts of marginalized rural areas for whom the (itinerant) health promotion services and support seem to be an important component of cohesion and equity [53–55]. The impact of design and intervention with community representatives is planned and further reflexion on follow-up of the healthy lifestyle assessment in rural (and urban) neighbourhoods is required, which is feasible using the tools in a reference site of the collaborative network European innovation partnership on active and healthy ageing (EIP on AHA) [28, 56–58].

## Conclusions

Revisiting our initial research aim to assess whether unhealthy lifestyles and environment in rural neighbourhoods are reflected into metabolic risks and health capability, we observed that NCD risk in overweight individuals (aged 55 to 74 years) was higher in men in all neighbourhoods; and metabolic risks were more associated to BMI and physical activity than diet (or sleeping habits). The qualitative research allowed us to uncovering seven environmental circumstances reflecting health needs, health expectations and health capability at community-level: economic development, built (and natural) environment, social network, health care, demography, active lifestyles, and mobility, which also underline the asymmetries among neighbourhoods. Notably, participants often reframed their narratives to express the benefits of living in rural areas. Natural resources and environment were pinpointed as the main value of rural well-being, with a particular focus on land use, biodiversity and connectivity with nature, as well as environmental quality. Our CBPR approach contributed for the active involvement of the local representatives and to adapt the health-related messages for older adults with limited literacy. The co-benefits from this co-designing community program and cross-disciplinary research provide further evidence to support people-centred approaches for pushing health and well-being at a broader social, health care and natural environment agenda in rural neighbourhoods.

## Supplementary Information

**Additional file 1: Table S1.** Evidence-based data by neighbourhoods’ type.

**Additional file 2: Table S2.** Characterization of the community environment needs by neighbourhoods’ type and self-assessment of neighbourhood’ satisfaction.

## Data Availability

Datasets used in the study are available from the corresponding author upon request.

## Abbreviations

AHA: Active and Healthy Ageing
BMI: Body Mass Index
CVD: Cardiovascular Disease
CBPR: Community-Based Participatory Research
DALYs: Disability-Adjusted-Life-Years
EIP: European Innovation Partnership
EIT Health: European Institute of Innovation and Technology for Health
HeaLIQs4Cities: Healthy Lifestyle Innovation Quarters for Cities and Citizens
ICTs: Information and Communication Technologies
IHME: Institute for Health, Metrics and Evaluation
mHLR: mobile Healthy Living Room
NCD: Non-Communicable Diseases
SBP: Systolic blood pressure
T2DM: Type 2 diabetes mellitus
WHO: World Health Organisation
