# Supplementary material for: Unhealthy lifestyles, environment, well-being and health capability in rural neighbourhoods: a community-based cross-sectional study
Source: BMC Public Health. 2021 Sep 6;21:1628. doi: 10.1186/s12889-021-11661-4 (PMC8422758; doi:10.1186/s12889-021-11661-4)
Supplement: Supplementary file 1 — Additional file 1: Table S1. Evidence-based data by neighbourhoods’ type. [file 12889_2021_11661_MOESM1_ESM.docx]

**Table S1.** Evidence-based data by neighbourhoods’ type.

|  |  |  |  |  |  |  |  |  |  |  |  |  |  |
| --- | --- | --- | --- | --- | --- | --- | --- | --- | --- | --- | --- | --- | --- |
|  | | Small Village | | | Parish Council | | | Municipality | | | Total | | |
|  |  | (n = 84) | | | (n=112) | | | (n=74) | | | (n=270) | | |
|  | | <54 | 55-74 | >75 | <54 | 55-74 | >75 | <54 | 55-74 | >75 | <54 | 55-74 | >75 |
| BMI, Kg/m^2^ | |  |  |  |  |  |  |  |  |  |  |  |  |
| Normal (<25) | |  |  |  |  |  |  |  |  |  |  |  |  |
| Female | | 3 (43%) | 3 (10%) | 5 (25%) | 4 (27%) | 4 (15%) | 6 (26%) | 7 (33%) | 8 (33%) | - | 14 (33%) | 15 (19%) | 11 (23%) |
| Male | | 1 (50%) | 1 (8%) | 5 (39%) | 2 (25%) | - | 2 (11%) | 2 (40%) | 1 (7%) | 1 (20%) | 5 (33%) | 2 (4%) | 8 (22%) |
| Overweight (25-30) | |  |  |  |  |  |  |  |  |  |  |  |  |
| Female | | 3 (43%) | 14 (48%) | 5 (25%) | 7 (47%) | 7 (27%) | 12 (52%) | 10 (48%) | 7 (29%) | 3 (60%) | 20 (47%) | 28 (35%) | 20 (42%) |
| Male | | - | 7 (54%) | 5 (39%) | 1 (13%) | 12 (55%) | 5 (28%) | 2 (40%) | 7 (50%) | 2 (40%) | 3 (20%) | 26 (53%) | 12 (33%) |
| Obesity (>30) | |  |  |  |  |  |  |  |  |  |  |  |  |
| Female | | 1 (14.3%) | 12 (41%) | 10 (50%) | 4 (27%) | 15 (58%) | 5 (22%) | 4 (19%) | 9 (38%) | 2 (40%) | 9 (21%) | 36 (46%) | 17 (35%) |
| Male | | 1 (50%) | 5 (39%) | 3 (23%) | 5 (63%) | 10 (46%) | 11 (61%) | 1 (20%) | 6 (43%) | 2 (40%) | 7 (47%) | 21 (43%) | 16 (44%) |
| Waist circumference, cm | |  |  |  |  |  |  |  |  |  |  |  |  |
| Female | | 86.8 (8.9) | 96.3 (7.2) | 99.1 (11.4) | 78.2 (5.9) | 99.2 (12.7) | 130.0 (182.4) | 84.2 (16) | 93.4  (15.2) | 97.6  (4.1) | 82.93 (13) | 96.3 (12.1) | 113.7 (127.5) |
| Male | | 90.9 (14.1) | 107.0 (7.2) | 98.7 (10) | 99.8 (9.8) | 104.0 (9.5) | 107.7 (8.1) | 92.1 (8) | 106.1 (11.8) | 106.8 (6.6) | 93.17 (6.7) | 105.4 (9.8) | 104.3 (9.6) |
| Illnesses or chronic diseases | |  |  |  |  |  |  |  |  |  |  |  |  |
| Heart disease (heart failure, ischemia or angina, arrhythmia) | | - | 7 (17%) | 10 (30%) | 1 (4%) | 7 (15%) | 14 (34%) | 2 (8%) | 3 (8%) | 4 (40%) | 3 (5%) | 17 (13%) | 28 (33%) |
| Peripheral vascular disease (arteries of the legs and feet or varicose veins) | | 1 (11%) | 3 (7%) | 3 (9%) | 1 (4%) | 4 (8%) | 2 (5%) | 4 (15%) | 3 (8%) | 1 (10%) | 6 (10%) | 10 (8%) | 6 (7%) |
| Respiratory disease (asthma, bronchitis, chronic obstructive pulmonary disease) | | - | 3 (7%) | 1 (3%) | - | 2 (4%) | 1 (2%) | - | - | - | - | 5 (4%) | 2 (2%) |
| Medication, nº per day | |  |  |  |  |  |  |  |  |  |  |  |  |
| 0 – 1 | | 6 (67%) | 5 (12%) | 6 (18%) | 14 (61%) | 16 (33%) | 4 (10%) | 20 (77%) | 12 (32%) | 1 (10%) | 40 (69%) | 33 (26%) | 11 (13%) |
| 2 – 5 | | 1 (11%) | 27 (64%) | 18 (55%) | 6 (26%) | 25 (52%) | 23 (56%) | 5 (19%) | 23 (61%) | 4 (40%) | 12 (21%) | 75 (59%) | 45 (54%) |
| > 5 | | 2 (22%) | 10 (24%) | 9 (27%) | 3 (13%) | 7 (15%) | 14 (34%) | 1 (4%) | 3 (8%) | 5 (50%) | 6 (10%) | 20 (16%) | 28 (33%) |
| Sleep habits, hours | |  |  |  |  |  |  |  |  |  |  |  |  |
| <5 | | 2 (22%) | 8 (19%) | 7 (21%) | 4 (17%) | 9 (19%) | 6 (15%) | 3 (12%) | 5 (13%) | 1 (10%) | 9 (16%) | 22 (17%) | 14 (17%) |
| 5 – 7 | | 6 (67%) | 18 (43%) | 15 (46%) | 10 (44%) | 25 (52%) | 17 (42%) | 7 (27%) | 14 (37%) | 4 (40%) | 23 (40%) | 57 (45%) | 36 (43%) |
| ≥7 | | 1 (11%) | 16 (39%) | 11 (33%) | 9 (39%) | 14 (29%) | 18 (44%) | 16 (62%) | 19 (50%) | 5 (50%) | 26 (45%) | 49 (38%) | 34 (41%) |
| Sleep quality | |  |  |  |  |  |  |  |  |  |  |  |  |
| Poor sleep (self-assessment) | | 5 (56%) | 17 (41%) | 15 (46%) | 10 (44%) | 16 (33%) | 13 (32%) | 4 (15%) | 12 (32%) | 4 (40%) | 19 (33%) | 45 (35%) | 32 (38%) |
| Waking up at night, nº times | |  |  |  |  |  |  |  |  |  |  |  |  |
| 0 – 1 | | 8 (89%) | 16 (38%) | 11 (33%) | 13 (57%) | 25 (52%) | 16 (39%) | 19 (73%) | 21 (55%) | 7 (70%) | 40 (69%) | 62 (48%) | 34 (41%) |
| 2 – 3 | | - | 15 (36%) | 18 (55%) | 6 (26%) | 15 (31%) | 21 (51%) | 6 (23%) | 12 (32%) | 2 (20%) | 12 (21%) | 42 (33%) | 41 (49%) |
| >3 | | 1 (11%) | 11 (26%) | 4 (12%) | 4 (17%) | 8 (17%) | 4 (10%) | 1 (4%) | 5 (13%) | 1 (10%) | 6 (10%) | 24 (19%) | 9 (11%) |
| Sleeping with medication | | 2 (22%) | 11 (26%) | 8 (24%) | 9 (39%) | 12 (25%) | 8 (20%) | 5 (19%) | 12 (32%) | 3 (30%) | 16 (27%) | 35 (27%) | 19 (23%) |
| Data are n (%), some percentages might not add to 100% due to optionality of not answering. | | | | | | | | | | | | | |
