# Supplementary material for: Unhealthy lifestyles, environment, well-being and health capability in rural neighbourhoods: a community-based cross-sectional study
Source: BMC Public Health. 2021 Sep 6;21:1628. doi: 10.1186/s12889-021-11661-4 (PMC8422758; doi:10.1186/s12889-021-11661-4)
Supplement: Supplementary file 2 — Additional file 2: Table S2. Characterization of the community environment needs by neighbourhoods’ type and self-assessment of neighbourhood’ satisfaction. [file 12889_2021_11661_MOESM2_ESM.docx]

**Table S2.** Characterization of the community environment needs by neighbourhoods’ type and self-assessment of neighbourhood’ satisfaction.

|  | Small Village | Parish Council | Municipality | Total |  |
| --- | --- | --- | --- | --- | --- |
|  | (n = 84) | (n=112) | (n=74) | (n=270) |  |
| Characterization of community environment (dimensions) |  |  |  |  |  |
| Community needs | | | | | |
| Economic development | 25 (30%) | 46 (41%) | 15 (20%) | 86 (32%) |  |
| Goods and services (cafes, grocery stores, restaurants, bank, book stores, shopping centers) | 21 (25%) | 23 (20%) | 4 (5%) | 48 (18%) |  |
| Employment | 2 (2%) | 10 (9%) | 8 (11%) | 20 (7%) |  |
| Financial investment, industry, industrial infrastructures | -- | 8 (7%) | 2 (3%) | 10 (4%) |  |
| Tourism | 2 (2%) | 5 (5%) | -- | 7 (3%) |  |
| Better salaries | -- | -- | 1 (1%) | 1 (0.4%) |  |
| Built environment | 15 (18%) | 48 (43%) | 10 (14%) | 73 (27%) |  |
| Infrastructures for cultural activities (folk activities, folk music, cinema or theatre) | 9 (11%) | 13 (12%) | 2 (3%) | 24 (9%) |  |
| Infrastructures for practicing exercise (gymnasium, swimming pool, tennis court and walking routes) | 1 (1%) | 14 (13%) | 3 (4%) | 18 (7%) |  |
| Infrastructures for green spaces (green parks and gardens, parks for children, campsite and river beaches) | 1 (1%) | 11 (10%) | 2 (3%) | 14 (5%) |  |
| Maintenance of regular infrastructures | 2 (2%) | 6 (5%) | 2 (3%) | 10 (4%) |  |
| Patrimony rehabilitation | 1 (1%) | 2 (2%) | -- | 3 (1%) |  |
| Social network | 16 (19%) | 52 (46%) | 13 (17%) | 81 (30%) |  |
| Group relationships and dynamics | 14 (17%) | 34 (30%) | 12 (16%) | 60 (22%) |  |
| Adult social care (day centers, nursing homes) | 2 (2%) | 18 (16%) | 1 (1%) | 21 (8%) |  |
| Healthcare | 8 (10%) | 31 (28%) | 3 (4%) | 42 (16%) |  |
| Primary healthcare services (doctors, health centers) | 8 (10%) | 28 (25%) | 3 (4%) | 39 (14%) |  |
| Pharmacy | -- | 2 (2%) | -- | 2 (1%) |  |
| Public transport to health services | -- | 1 (1%) | -- | 1 (0.4%) |  |
| Demography | 11 (13%) | 21 (18%) | 5 (6%) | 37 (14%) |  |
| More people (young people, children) | 10 (12%) | 16 (14%) | 4 (5%) | 30 (11%) |  |
| Social pressure to maintain schools, kindergartens | -- | 5 (5%) | 1 (1%) | 6(2%) |  |
| Active lifestyle | 4 (5%) | 22 (20%) | 9 (12%) | 35 (13%) |  |
| Lifelong learning and digital inclusion | 2 (2%) | 11 (10%) | 4 (5%) | 17 (6%) |  |
| Physical activities and exercise | 2 (2%) | 6 (5%) | 2 (3%) | 10 (4%) |  |
| Cultural activities | -- | 5 (5%) | 3 (4%) | 8 (3%) |  |
| Mobility | 15 (18%) | 8 (7%) | 7 (10%) | 30 (11%) |  |
| Accessible public transport | 12 (14%) | 8 (7%) | 5 (7%) | 25 (9%) |  |
| Accessible walking routes | 3 (4%) | -- | 2 (3%) | 5 (2%) |  |
| None | 34 (41%) | 27 (24%) | 31 (42%) | 92 (34%) |  |
| Self-assessment of satisfaction |  |  |  |  |  |
| I like to live in my neighbourhood | | | | | |
| Anything | 1 (1%) | 3 (3%) | 1 (1%) | 5 (2%) |  |
| Little | 4 (5%) | 3 (3%) | -- | 7 (3%) |  |
| Neither like nor dislike | 8 (10%) | 5 (5%) | 8 (11%) | 21 (8%) |  |
| Very | 52 (62%) | 60 (54%) | 45 (61%) | 157 (58%) |  |
| Very much | 19 (23%) | 41 (37%) | 20 (27%) | 80 (30%) |  |
| I consider my neighbourhood is a good place to grow old | |  |  |  |  |
| Strongly disagree | 1 (1%) | -- | 2 (3%) | 3 (1%) |  |
| Disagree | 6 (7%) | 9 (8%) | 6 (8%) | 21 (8%) |  |
| Neither agree nor disagree | 12 (14%) | 11 (10%) | 7 (10%) | 30 (11%) |  |
| Agree | 44 (52%) | 61 (55%) | 37 (50%) | 142 (53%) |  |
| Totally agree | 21 (25%) | 31 (28%) | 22 (30%) | 74 (27%) |  |
| I would live elsewhere if I could |  |  |  |  |  |
| Strongly disagree | 35 (42%) | 31 (28%) | 8 (11%) | 74 (27%) |  |
| Disagree | 22 (27%) | 35 (31%) | 25 (34%) | 82 (30%) |  |
| Neither agree nor disagree | 11 (13%) | 14 (13%) | 11 (15%) | 36 (13%) |  |
| Agree | 10 (12%) | 20 (18%) | 13 (18%) | 43 (16%) |  |
| Totally agree | 5 (6%) | 12 (11%) | 17 (23%) | 34 (13%) |  |
| Data are n (%), some percentages might not add to 100% due to optionality of not answering. | | | | | |
